# Emerging Trends in Bioavailability and Pharma-Nutraceutical Potential of Whey Bioactives

**DOI:** 10.1155/2024/8455666

**Published:** 2024-04-10

**Authors:** Adhithyan T. Pillai, Sonia Morya, Ladislaus Manaku Kasankala

**Affiliations:** ^1^Department of Food Technology and Nutrition, School of Agriculture, Lovely Professional University, Phagwara 144411, Punjab, India; ^2^Department of Food Science and Nutrition, Tanzania Food Science and Nutrition, Dar es Salaam, Tanzania

## Abstract

Whey, a component of milk and a useful by-product of the dairy industry's casein and cheese-making, has been used for generations to augment animal feed. It contains a range of proteins, including *α*-lactalbumin, *β*-lactoglobulin, bovine serum albumin, heavy and light chain immunoglobulins, lactoferrin, glycomacropeptide, and lactoperoxidase. Whey proteins exhibit great potential as biopolymers for creating bioactive delivery systems owing to their distinct health-enhancing characteristics and the presence of numerous amino acid groups within their structures. Whey has considerable factors such as antitumor, anti-inflammatory, antihypertensive, hypolipidemic, antiviral, and antibacterial properties in addition to chelating. The global market of whey protein stood at USD 5.33 billion in 2021, with a projected compound annual growth rate of 10.48% spanning the interval from 2022 to 2030. The escalating demand for whey protein is intrinsically linked to the amplifying consciousness surrounding healthy lifestyles. Notably, protein supplements are recurrently endorsed by fitness and sports establishments, thereby accentuating the focal point of customers toward whey protein. This review focuses on nutritional composition, whey bioactives, and their bioavailability with potential health benefits.

## 1. Introduction

Cow's milk, a nutritious food, is frequently consumed by people all across the world. It is one of the many dietary sources that influence our bodies' biochemical processes, control the metabolic development and operation of various organs, and offer immunity [1]. Bioactive oligosaccharides, immunoglobulins, fats/lipids, proteins, and peptides are only a few of the physiologically active components that milk carries on a regular basis to fight off illnesses and infections [2]. It has a wide range of bio-functional properties and is a naturally occurring, plentiful source of balanced nutrients. These characteristics are the result of the presence of milk proteins and peptides, which promote growth, build muscles, and show a positive effect on health that goes far beyond the basic nutrition. Various enterprises use milk proteins in culinary applications because of their diverse functional qualities as well [3].

Whey is a result of the process used to make cheese in the dairy industry. Only 20% of milk is made up of whey. In addition, there are substances such as lactose, protein, fat, and water. Whey is the liquid that is produced when milk coagulates and is strained to make cheese. Sweet whey, having a minimum pH of 5.6, is obtained during the production of cheeses like cheddar that are coagulated with rennet. Acid-coagulated cheeses like cottage cheese are made with whey that has a pH of no more than 5.1 [4–6]. A large cheese factory may create more than 1 million liters of whey every day. Nine liters of whey are produced as the residue of each kilogram of cheese produced. When whey is not consumed by people, it is supplied to pigs and various other cattle, used as a fertilizer, or discarded [7]. Whey has been used for generations to augment pig feed, and the development of computerised equipment has made it possible to give whey and various other liquid feeds to weaned piglets and nursing cows more precisely. Previously, there was a huge production of cheese in the USA and whey was directly disposed into the water bodies as waste product after the production process of cheese. Four thousand liters of whey, the by-product of a tiny creamery, possesses the damaging strength of 1,900 people's sewage. Whey is an extremely hazardous contaminant that has a biological oxygen requirement of 35–45 kg/L [8].

Several studies have been carried out on whey during the industrial periods in the modern era and numerous aspects regarding whey and its nutritional composition have been discovered. Whey is currently used for its bioactive components and is acknowledged as a possible source of nutrients [2, 9]. It has a substantial connection to the dairy sector and is employed in a variety of commercial food product applications due to its highly nutritious content. In general, liquid whey obtained from cheese-making has 94.3% water, and 50% total solids, from which 4.3% lactose, 0.8% whey proteins, 0.5% minerals, and 0.1% fat can be extracted [10]. Beta-lactoglobulin (*β*-LG), alpha-lactalbumin (*α*-LA), bovine lactoperoxidase (LP), immunoglobulins (IGs), bovine lactoferrin (BLF), bovine serum albumin (BSA), and minor levels of glycomacropeptide (GMP) are the main components of whey proteins. However, depending on the type of whey-acid or sweet as well as the kind of milk, bovine, ovine, or caprine, the type of cow feed, the stage of lactation, and the method of processing the milk, the protein concentration in whey differs [11]. Thus, as whey contains nutrients that are very much beneficial for human health, it is now thought to be a suitable consumable for humans but not only as animal feed.

All the essential amino acids such as histidine, isoleucine, leucine, lysine, methionine, phenylalanine, threonine, tryptophan, and valine are present in high-quality proteins known as bovine whey proteins. These proteins also contain bioactive peptides, which facilitate easy digestion [10]. The total content and bioavailability of whey were estimated using the inductively coupled plasma mass spectroscopy method in order to assess the potential risk associated with increased daily consumption of whey products like whey protein, etc. According to the findings, the most common components were Na, K, Ca, and Mg [12]. The average basic bioavailable fraction in the intestine is 64% as opposed to 45% in the stomach. Nineteen of the studied elements (the majority) had gastric bioavailability values of more than 60%, with aluminium having the lowest value (37%) and cobalt having the highest value (60%) (76%) [13]. Amino acid molecules containing branched chains have been proven to be essential for keeping muscle cells healthy, with their effects being felt most strongly in the areas of metabolism, protein synthesis, mitochondrial biogenesis, and redox equilibrium [14].

In short, whey is a milk-derived protein complex, promoted as a functional food that has multiple health advantages. The whey biological components like *β*-LG*β*-lactoglobulin, glycomacropeptide, *α*-LA*α*-lactalbumin, lactoferrin, and immunoglobulins exhibit a range of immune-boosting characteristics [15]. Whey has antitumor, anti-inflammatory, antihypertensive, hypolipidemic, antiviral, and antibacterial properties in addition to chelating [16]. The intracellular metabolism of cysteine, an amino acid, to glutathione, a powerful intracellular antioxidant, is the main mechanism through which whey is believed to provide its benefits. Numerous clinical studies have shown the use of whey in the resistance of diseases like cancer, hepatitis B, osteoporosis, HIV, cardiovascular disease, and as an antibacterial agent [17].

This review focuses on the different nutritional compositions, bioactives, applications, and bioactives bioavailability of whey. The uses and applications of acid whey are reviewed, as well as any potential health advantages. There is a thorough discussion of the proximal makeup of acid whey. Some of the whey constituents and their concentrations in cow milk have been mentioned in Table 1.

## 2. Description of Selected Bioactive Whey Protein Components and Their Health Effects

**Figure 1:**
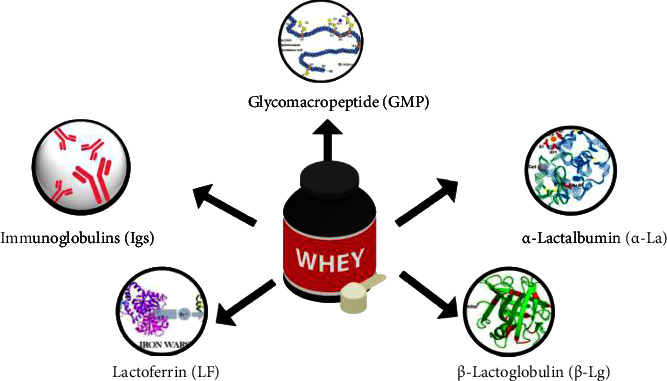
Different proteins in milk whey.

Whey, a component of milk and a useful by-product of the dairy industry's casein and cheese-making, contains a range of proteins, including lactalbumin (LA), lactglobulin (LG), bovine serum albumin (BSA), heavy chain and light chain immunoglobulins (IGs), lactoferrin (LF), glycomacropeptide (GMP), and lactoperoxidase [20] as shown in Figure 1.

All of these elements are advantageous to human health and are covered in detail.

### 2.1. Alpha-Lactalbumin (*α*-La)

Alpha-lactalbumin (*α*-La) is among the most prevalent nutritious protein compounds in both cow's and human milk. It comprises between 20 and 25% whey proteins and offers a broad range of highly advantageous amino acids, consisting of a convenient source of branched-chain and essential amino acids [21].

A coenzyme called *α*-lactalbumin is required in the mammary gland to produce lactose. The advantages of *α*-La for health have long been known to nutritionists and food scientists, and a recent study indicates that this protein may have positive effects via (1) the intact whole molecule, (2) partially hydrolyzed protein peptides, and (3) fully digested protein amino acids [22]. Tryptophan and cysteine, two crucial amino acids that serve as the building blocks for glutathione and serotonin, respectively, are abundant in *α*-La. According to research, consuming *α*-La orally may improve stress management [8]. Tryptophan, which is plentiful in *α*-La and raises serotonin levels, can lower chronic stress, by acting as a substrate essential for good mood. The proliferation of mammary epithelial tissue and healthy rat kidney tissue in culture has been shown to be reduced by human *α*-La, but not fibroblasts [19]). The idea that *α*-La acts as a feedback blocker of mammary cell growth during breastfeeding is supported by the observation that preparations of *α*-La from a variety of mammalian species reduced the growth of cultured mammary epithelial cells, as well as other types of cells wholly or partially. An example of an immunostimulant is *α*-La. In addition, it boosts the synthesis of lacto immunopeptides and interleukin-1 (IL-1) by cultured ovine Broncho alveolar lavage macrophages [1]. It also encourages the growth of human blood cells in culture.

### 2.2. Glycomacropeptide (GMP)

Whey contains glycomacropeptide (GMP), the glycosylated version of caseinomacropeptide (CMP), which is produced by the renin-mediated cleavage of k-casein and precipitation of casein. When the casein gets coagulated by bringing down the pH to 4.6, acid whey is created, but it lacks the presence of glycomacropeptide (GMP). In sweet cheese whey, GMP makes up 15-20% of the total protein, which is a rich protein [23].

Due to the nature of its glycoproteins, GMP has special nutritive and physico-chemical characteristics. GMP is a beneficial addition to the diets of people with liver illness because it is low in methionine and contains a lot of branched-chain amino acids. GMP is appropriate for people with phenylketonuria (PKU) because it does not contain phenylalanine [24]. The whey proteins lactoglobulin and lactalbumin include phenylalanine and contaminate GMP when it is separated from cheese whey. Thus, each gram of protein in commercial GMP includes 2.0 to 5.0 mg of phenylalanine. Since GMP comprises between two and three times the amount as the large neutral amino acids (LNAAs) threonine, isoleucine, and valine as other dietary proteins, it must be supplemented with the essential amino acids to give a full protein intake for people with phenylketonuria (PKU) [25].

GMP's high sialic acid content has been shown in animal research to have favorable impacts on brain development and learning ability. By encouraging the satiety hormone cholecystokinin (CKK), which regulates dietary intake and digestion in both the animal and human duodenum, to be produced, it has been demonstrated that GMP limits gastric secretions and decreases stomach motility [26]. In numerous studies, GMP has been found to reduce food intake and boost satiety. In a recent investigation, healthy volunteers who depended on whey with GMP ate around 10% fewer calories at lunch than those who consumed whey without GMP [27]. When opposed to proteins that are digested more slowly, such as casein, whey protein's rapid digestion and absorption cause a quick rise in the levels of the hormone's insulin, glucagon-like peptide-1, cholecystokinin, and plasma amino acids. Glycomacropeptides restrict stomach digestion and heighten satiety by triggering cholecystokinin, the hormone in the intestinal cells that regulate the energy and food adsorption by the cells. In addition, it promotes bowel movement and gall bladder contraction, controls stomach emptying, and promotes the release of pancreatic enzymes [28]. GMP-containing commercial medications for hunger management and weight management have been made available to the public in recent years. GMP, on the other hand, has been shown to enhance the proliferation of bifidobacterium, indicating that it might be involved in the regulation of the gut microbiota [29]. When opposed to proteins that are digested more slowly, such as casein, whey protein's rapid digestion and absorption cause a quick rise in the levels of the hormones insulin, glucagon-like peptide-1, cholecystokinin, and plasma amino acids. Glycomacropeptides inhibit stomach digestion and increase satiety by causing intestinal cells to release the hormone cholecystokinin, which regulates energy and food intake. In addition, it modulates stomach emptying, promotes pancreatic enzyme release, and stimulates bowel motility in addition to stimulating gall bladder contraction [30]. GMP-containing commercial medications for hunger control and weight management have recently been released in the market. GMP, on the other hand, has been demonstrated to promote the growth of bifidobacterium, indicating that it might be involved in the regulation of the gut microbiota [31].

### 2.3. Beta-Lactoglobulin (*β*-LG)

Although it is rarely seen in human milk, *β*-LG makes up approximately 50% of all whey proteins in cow milk, making it the most common kind [32]. *β*-LG is a flexible component material for a variety of food and biochemical applications due to its wide range of functional and nutritional qualities.

*β*-LgLG is mainly responsible for the mitigation of hypocholesterolemia, antiviral activity, and the suppression of pathogen adhesion. *β*-LG binds to retinol, triglycerides, and long-chain fatty acids in preruminant calves, improving their intestinal absorption. Furthermore, *β*-LG has been demonstrated to be a trustworthy source of peptides with a variety of bioactivities, including antibacterial, antihypertensive, antioxidant, anticarcinogenic, hypocholesterolemia, opioid, immunomodulatory, and other metabolic characteristics [33]. The antihypertensive peptides *α*-lactorphin and *β*-lactorphin, which are derived from the milk whey proteins *α*-LA*α*-lactalbumin and *β*-lactoglobulin, respectively, by enzymatic proteolysis by stomach and pancreatic enzymes, are found in the whey fraction of milk protein. A tetrapeptide created from lactoglobulin and lactosin demonstrated a notable antihypertensive effect in the spontaneous hypertensive rate (SHR). A single dose of proteinase K-digested cheese-derived whey decreased the blood pressure in SHR. The digested peptide with the strongest antihypertensive effect was found to be derived from *β*-LG. The peptides mentioned above are all ACE inhibitors or angiotensin-converting enzyme inhibitors. ACE inhibition is assumed to be the main mechanism of action of whey-derived peptides' antihypertensive impact. The rennin-angiotensin system, of which ACE is an essential part, regulates the salt and water balance as well as arterial blood pressure. Bradykinin, a robust vasodilator, is degraded by ACE, while angiotensin I is converted to angiotensin II, a potent vasoconstrictor [34].

Sadly, *β*-LG is the main component of cow's milk that causes milk allergy. To lessen antigenicity, *β*-LG was conjugated with acidic oligosaccharides. The conjugates may be helpful in lowering Th2-mediated allergies since they reduced the T-cell response in mice following immunization that was largely Th1-mediated. When taken internally, recombinant Lactococcus lactis, a bacterium that expresses bovine *β*-LG elicited a particular response, suggesting that probiotics expressing *β*-LG may be helpful in the treatment of food allergies [35].

### 2.4. Immunoglobulins (IGs)

As a portion of the total bovine whey proteins, immunoglobulins make up roughly 10-15% of the milk's whey fraction and their concentration varies from milk to milk (Table 2). The majority of IGs are made up of a total of four polypeptide chains, with each one containing two identical heavy chains joined together by covalent and noncovalent disulfide bridges. When people drink whey-carrying IGs, which is desired, cow milk antibodies remain extremely susceptible to peptic digestion [39].

Humans are well shielded from pathogens, viruses, and parasites by antibodies made from cow's milk. Antibodies found in whey protein each have a specific function in the immune system [40]. Immunoglobulins such as IgG1, IgG2, IgM, and IgA are present in whey. Only bacteria and viruses contain IgM, a kind of antibody that responds swiftly to an antigen. IgG1 and IgG2 target viruses and other toxins after IgM. IgA is crucial for viral defense and prevents bacterial adherence.

The advantage of passive administration of these antibodies is evident when gastrointestinal distress occurs (for instance, in the case of AIDS). The epithelium is a robust chemical barrier in addition to serving as a strong physical barrier against the entry of foreign chemicals. In the presence of secretory IGs, antigens bind in the lumen [41]. Without this binding, the antigen would possibly cause disease by penetrating the barrier and encrusting itself inside the enterocyte. Instead, it prevents the antigen from adhering to or penetrating the intestine's epithelial layer. Therefore, passively delivered IGs might aid in lessening gastrointestinal antigen adherence [42].

Immune milk formulations are beneficial in avoiding human and animal infections caused by pathogenic microorganisms such rotavirus, *Candida albicans*, *Escherichia coli*, *Shigella flexneri*, *Clostridium difficile,* and *Streptococcus mutans*, according to numerous clinical studies. These formulations have been shown to be effective against *Helicobacter pylori* and *Cryptosporidium parvum* [43]. A few commercialized immune-boosting milk products are offered in various markets, but international commercialization has been hampered by the products' murky regulatory position in many of these countries. Research focusing on the development of immune-boosting milk products to counter various illnesses promises to be an intriguing future research topic given the global increase in antibiotic-resistant strains causing endemic hospital infections [44].

### 2.5. Lactoferrin (LF)

Lactoferrin (LF), an iron-binding glycoprotein present in milk's whey and colostrum, is a nonenzymatic antioxidant. One polypeptide chain with two ferric ion–binding sites makes up the structure of whey LF. Antibacterial, anti-inflammatory, antioxidant, anticancer, and immune-regulatory actions of LF have been demonstrated in Table 2 [36, 37], which includes a mention of lactoferrin (%).

The oral consumption of LF has been linked to a number of positive impacts on both human and animal health, according to a number of researches conducted on both species over the last three decades. In animal studies, it has been demonstrated that LF given orally inhibits the growth and translocation of some gut bacteria while having no impact on intestinal bifidobacterium. By giving LF and lactoferricin orally, infections with *Toxoplasma gondii*, *Tinea pedis*, *Candidiasis,* and *Helicobacter pylori* are less common, and the clinical symptoms of influenza virus infection are also prevented. By lowering endotoxin shock mortality, colitis, arthritis, drug-induced intestinal inflammation, and iron deficiency anemia, orally consumed LF and related chemicals also enhance nutritional status [45].

A new study found that giving mice oral LF therapy altered the function of bone cells and promoted bone development. Babies who receive oral bovine LF preparations have been observed to have higher levels of blood ferritin and fecal bifidobacterium while having lower ratios of *streptococcus*, *enterobacteriaceae,* and *clostridium* [46]. In a recent clinical study, it was found that over a period of 12 months for healthy newborns, LF was related to lower infections of the respiratory tract and higher hematocrits than normal infant formula in the control group. Combining LF with triple therapy has been proven to boost the rate of *H. pylori* gastritis eradication [47].

## 3. Applications of Whey and Its Bioactive Components in Various Fields

### 3.1. Pharmaceutical and Nutraceutical Application of Whey Bioactives

Along with their typical use, whey and its derivatives have a variety of other uses. Whey has a variety of medicinal uses in addition to its other biological applications.

In addition to bovine serum albumin, alpha-lactalbumin, beta-lactoglobulin, immunoglobulins, lactoperoxidase, lactoferrin, and whey protein contain a significant amount of other bioactive substances. Due to their bioactive properties, these proteins have been employed as components in pharmacological, nutraceutical, and cosmeceutical applications [48]. Numerous studies suggested that all bioactive peptides have a number of health advantages, including boosting our immune system [49], avoiding infections as a matter of LF's antiviral activity [50], oxidative stress reduction and infection caused by human immunodeficiency virus (HIV) [51], and cancerous activity that lessens anxiety, which reduces blood pressure [52], protection against hepatitis [53], cardiovascular diseases, and osteoporosis.

### 3.2. Antioxidant Properties

**Figure 2:**
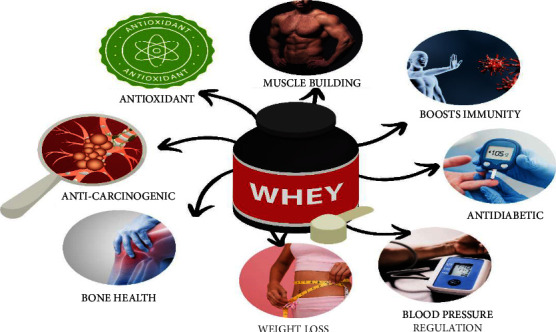
Therapeutical application of whey.

Numerous conditions, including cancer, atherosclerosis, cystic fibrosis, aging, diabetes, and other degenerative diseases, occur as a consequence of a process called oxidative stress in the body. According to Reference [54], whey protein demonstrates antioxidant activity by decreasing the negative effects of stress factors, which are considered a precursor to the antioxidant glutathione (Figure 2). In cystic fibrosis patients, supplementing with compressed whey (20 g/day) for a month reduced the serum level of C-reactive protein [55]. The cysteine is found in whey protein, which is generally associated with the improved antioxidant activity of individuals. It is usually found in the form of cystine in the whey protein components. The antioxidant effects of milk are provided by the amino acid content. Depending on the type of milk, it varies, such as cow, goat, or buffalo milk. The % of amino acids found in different milks is displayed in Table 3, [48].

It was discovered that the alcalase enzymes used to generate whey protein hydrolysates contained peptide fragments, P4c and P4 (pentapeptide with the amino acids Val-His-Leu-Lys-Pro). These peptides displayed antioxidant activity by significantly reducing the exposure of human lung fibroblast MRC-5 cells to hydrogen peroxide [56]. Rats were used to investigate the anti-inflammatory effects of a diet (MHN-02) prepared with antioxidants and whey peptides [57]. It was demonstrated that the rats fed this food had a greater chance of surviving (90%) than the rats fed the control diet (55%). The reason for this was that the MHN-02 diet group had lower levels of degenerative lesions and higher levels of superoxide dismutase activity, which breaks down superoxide radicals into hydrogen peroxide and oxygen.

Whey protein derivatives can lessen neuro-system disorders and increase glutathione synthesis in neurons. When Caco-2 cells from human epithelial colorectal cancer were exposed to H2O2, the production of IL-8 and reactive oxygen species (ROS) was reduced by both whey protein concentrates and native hydrolysates containing antioxidant and anti-inflammatory peptides. The whey protein hydrolysis products from isolates may be more effective at reducing oxidative stress and inflammation in intestinal cells, as evidenced by the fact that the benefit of whey protein isolated therapy was much stronger [58].

It was also demonstrated that these activities increased during hyperbaric therapy. According to one study, rats given high iron doses were given a whey protein meal or a placebo to see how it affected their levels of oxidative stress. The test animals' lipid peroxidation increased and their capacity to scavenge free radicals decreased after 6 weeks [59]. As opposed to the control group (iron overload), rats fed a whey protein diet showed greater blood levels of glutathione. This revealed that whey proteins may be able to change the DNA damage caused by elevated iron levels and lessen ROS in cells [60]. *Pseudomonas aeruginosa* is one of the recognized microorganisms that cause pulmonary infection and lung colonization, which lead to breathing problems [61]. Rats fed a pressurized whey protein diet exhibited a reduction in oxidative stressors, inflammation, and lung damage, according to studies on the importance of whey protein in decreasing pulmonary infection by Kishta and his team [62]. One hypothesis was that the peptides would protect the proteins in the airways from oxidation while also causing leucocytes to become active in the fight against infections. Whey protein hydrolysate was found to increase levels of antioxidant enzymes such as catalase, glutathione peroxidase, and superoxide dismutase while decreasing the production of oxidative biomarkers like phosphatase, creatinine, and glutathione pyruvate transaminase in mice with paracetamol-induced hepato-nephrotoxicity [63]. In addition, the ferrous chelating and DPPH radical-scavenging potential of peptides synthesized from chymotrypsin-hydrolyzed whey proteins are superior to those of whey protein isolates [64].

### 3.3. Antidiabetic Properties

Whey protein is a powerful source of numerous advantages. It helps treat a variety of disorders because of its antitoxin, antibacterial, and immunomodulating qualities. Whey and its bioactive provide a variety of health benefits. As shown in Figure 2, whey is extremely helpful in the treatment of diabetes. Diabetes is a major health condition that can lead to issues including vision loss, issues with healing, blood flow limits, etc. [65]. The prevention of type 2 diabetes can also be greatly aided by the use of hypoglycemic chemical drugs in conjunction with a restricted diet. In addition to improving muscle growth and promoting the release of numerous hormones, the addition of nutritional supplements to whey has been proven to have antidiabetic effects by lowering blood serum glucose levels [55]. The presence of cysteine has been found to aid in the management of glycemia and, as a result, avoid inflammation in diabetics [66]. In a study, whey proteins were found to lessen diabetic inflammatory conditions and wounds by reducing the production of cytokines, which are the cause of inflammation and manifest themselves throughout the body's tissues [65].

In individuals with type 2 diabetes, the addition of whey protein derivatives (isolate and hydrolysate) to a diet high in fat was observed to enhance the production of insulin and reduce postprandial triglyceride responses [66]. When the role of whey protein in lowering glucose levels was examined, it was found that the protein had a significant impact on C-peptide levels, plasma glucose, and insulin. The levels of PYY and GLP-1, however, were elevated, indicating that both insulin-dependent and insulin-independent pathways can reduce postmeal glycemia when whey protein is taken before a meal [67].

### 3.4. Anticancer

Several studies have demonstrated that whey protein consumption has positive effects on cancer patients. According to reports, whey protein hydrolysates have a stronger anticancer effect than other whey protein types. In comparison to the rats in the control group, which received whey protein that had not been hydrolyzed, the colon cancer-bearing rats given whey protein hydrolysate displayed decreased macroscopic and microscopic tumor growth [68]. As seen in Figure 2, whey protein was examined for its anticancer effects on the B16F10 melanoma cell type. It was found that the presence of whey protein isolate greatly raised the expression of caspase-3 in the medium [69]. According to Teixeira et al. [68], caspase-3 is well known for being a key mediator of apoptotic cell death. Lean body mass, physical function, and general quality of life of a 48-year-old Caucasian woman receiving chemotherapy increased when whey protein at a dosage of 10 g (three times daily) was paired with a weekly injection of testosterone enanthate [70]. In another study, it was discovered that whey protein hydrolysate can shield rat pheochromocytoma PC 12 cells from oxidative damage. A dosage level of 100–400 g hydrolysate/mL led to a 20-30% increase in cell viability when compared to cells that were treated with an infusion of H_2_O_2_. This implies that whey protein hydrolysates may possess antioxidant properties [1].

### 3.5. Immunomodulatory Activity

According to research [71], concentrates of whey protein derivatives can increase innate mucosal immunity and shield the body from immunological disorders. Atopic dermatitis, a condition marked by swollen, scaly skin and itchy rashes, is an increasing public health concern around the globe, especially for infants. According to a recent meta-analysis, babies who consumed hydrolyzed whey protein instead of normal cow's milk experienced fewer atopic dermatitis symptoms. These results suggested that whey protein-rich diets may aid in preventing the development of atopic dermatitis in neonates [72].

Patients received a daily dosage of 20 g of whey protein isolates to study its bioactive effects on psoriasis, a skin condition marked by dry scales, thick skin, and red patches. Following the inflammation brought on by psoriasis, the glutathione level increased with the subsequent consumption of whey protein before falling [71].

### 3.6. Physiological Effect of Peptides

**Figure 3:**
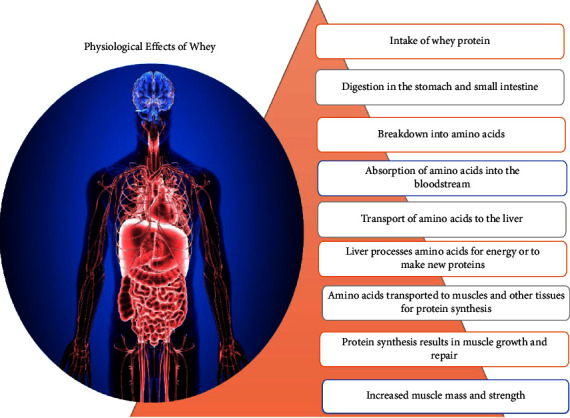
Physiological effect of whey proteins inside the human body.

Whey contains proteins that are often hydrolyzed by certain enzymes like pancreatic or stomach protease, which usually break down the main protein typically present in whey. In general, enzymes from plants and biological sources, including microorganisms, have a better potential to hydrolyze other enzymes and aid in the reduction of these bioactives.  Figure 3 discusses whey's physiological effects.

In addition to other minor proteinaceous components, whey proteins comprise *β*-LG, immunoglobulins, *β*-LA, BSA, lactoperoxidase, and bovine lactoferrin (BLF), as well as glycomacropeptide (GMP), which is created from k-casein in the first step of enzymatic cheese-making. According to Madureira et al. [73], these proteins have significant nutritional and biological qualities that can help promote health and aid in the prevention of diseases and health issues. In addition to other minor proteinaceous components like glycomacropeptide (GMP), which is formed from -casein in the first step of enzymatic cheese-making, the whey proteins include -LG, -LA, immunoglobulins, BSA, bovine lactoferrin (BLF), and lactoperoxidase. According to Madureira et al. [73], a whey protein shows significant nutritional quality and biological qualities that can help promote health and avoid diseases and health issues.

### 3.7. Therapeutic Applications in COVID-9 Infection

According to a recent study [74], consuming nutritional syrup made from liposomal bovine albumin (32 mg/10 mL) and vitamin C (12 mg) is an effective pretreatment for managing an emergent COVID-19 infection. According to another study, the SARS-CoV-2 protein receptor–binding area would specifically interact with the bovine-IgG fraction, indicating that the IgG-enrich fraction is extremely likely to be successful in neutralizing the SARS-CoV-2 virus [75].

Blood pressure and hypertension are significantly regulated by the rennin-angiotensin system (RAS). The maintenance of blood pressure homeostasis depends on the two primary RAS molecules, angiotensin-converting enzyme I (ACE) and ACE2. The ACE2 receptor is used by the coronavirus responsible for severe acute respiratory syndrome type 2 (SARS-CoV-2) to enter cells [75]. Numerous studies have found a link between the usage of ACE inhibitors and a decreased incidence of COVID-19, despite some controversy. Natural peptides made from whey show dual inhibitory effects on both ACE and ACE2. These ACE inhibitory peptides' dual activity set them apart from synthetic medications like lisinopril and captopril, which were not demonstrated to inhibit ACE2 activity and could be a therapy option for COVID-19 [76].

**Figure 4:**
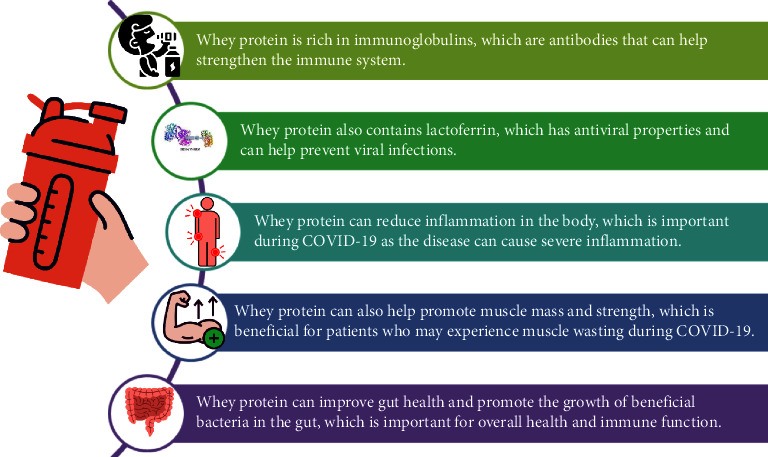
Therapeutic application of whey protein during COVID-19.

According to these researches, whey and its bioactives are extremely beneficial in preventing and treating COVID-19 infection. Human whey protein has been used therapeutically in the applications listed in Table 4 and Figure 4.

### 3.8. Bioavailability of Whey Proteins

Bioavailability is a statistic that describes the nutritional content's effectiveness in its simplest form. The body's ability to digest and recognize particular amino acids for later use is the most crucial factor in evaluating the nutritional value of a protein source [80]. The bioavailability score of a protein serves as a measure of nutritional potential based on the importance of the amino acid profile and the rate of active nitrogen-based components' absorption during digestion. If a protein has a high bioavailability score, the body may utilise it as a source of raw materials more frequently [81]. The bioavailability potential of whey is the data point that we must typically focus on. The “bioavailability” component also depends on a variety of additional factors, many of which are unrelated to the products themselves. High Bioavailability Potential: When two individuals consume the same quantity of whey protein powder under the same circumstances, individual A absorbs 80% of the amino acids whereas individual B only absorbs 10% [82]. Low Bioavailability Potential: When two individuals consume the same quantity of whey protein powder under the same circumstances, individual A absorbs 16% of the amino acids whereas individual B only absorbs 2% [83]. As seen in the differences between person A and person B in both cases, we can infer that the variance in absorption capacity in these scenarios has a lot to do with the intrinsic features of the individual. The first example's whey protein has a very high potential for bioavailability, though. Even if the user has excellent gut health, there is not much bioavailability potential in the second example due to the poor food quality [84].

There are two main methods for determining the bioavailability of whey protein isolate and the majority of proteins. It is crucial to consider the wide range of bioavailability variations between the many protein types and even within the same type, depending on the protein's quality, as not all proteins are made equal. Depending on recognized methods for calculating bioavailability, there are variances for each protein type. There is a general range of nutritional value for each protein type. Bioavailability charts will provide several food items together with a corresponding rating for bioavailability [85]. The (Protein Digestibility Corrected Amino Acid Score) PDCAAS and (Digestible Indispensable Amino Acid Score) DIAAS measures are the ones that are most frequently used to measure “bioavailability”.

### 3.9. Protein Digestibility Corrected Amino Acid Score (PDCAAS)

PDCAAS stands for “Protein Digestibility Corrected Amino Acid Score.” For the past 25 years, the PDCAAS method has been the accepted global standard for determining protein bioavailability scores. This procedure helped dairy protein powders gain widespread acceptance and popularize grass-fed dairy protein [86].

It is a fundamental concept that focuses on the production of food, particularly the essential amino acids (EAA, which the body cannot create on its own). The method compares EAA's chemical makeup to what the body needs to sustain a child between the ages of two and five as they develop and grow. Using feces, the protein is once again examined before consumption [87]. The bioavailability of proteins is assumed to be determined by the variation in amino acid contents between samples. Whey protein tops the list of numerous protein sources, receiving a perfect PDCAAS rating. PDCAAS (bioavailability value) of 1.0 is the most that a protein supply can attain, which is unfortunate because there is not much resolution at the top. According to Rodríguez Arzuaga et al. [88], the value of 1.0 shows that the second measurement of the EAAs concentration from the feces is significantly lower than the amino acids measured before eating.

The PDCAAS was tremendously beneficial to dietitians and the general public for a very long time. However, it is now understood that the PDCAAS's flaws significantly harm the credibility of the scoring system. These issues have a big effect on the value proposition of various protein sources. Digestible Indispensable Amino Acid Score is the most recent and practical method for determining bioavailability through dietary quality evaluation [89].

### 3.10. Digestible Indispensable Amino Acid Score (DIAAS)

The creation of the DIASS scoring approach addresses the shortcomings of the traditional PDCAAS. The foundation of the DIAAS is the notion that the ability to digest protein and utilise its nitrogen-based macronutrients does not always equate to the digestibility of certain essential amino acids for human nutrition. Using a method that creates a score based on each of the dietary necessary amino acids independently for true digestibility is preferred and more accurate [90]. The DIAAS model removes the second sample before the meal is exposed to fermentation in the large intestine, in contrast to the PDCAAS model. The fermentation in the large intestine is regarded to be a primary factor in inaccurate bioavailability assessments because of the nitrogen depletion that occurs there [91]. The term “digestible indispensable amino acid score” (DIAAS) refers to the ratio of an amino acid in a meal (mg/g of protein) to that same amino acid in a reference pattern constructed from age-specific amino acid demands. The essential amino acid that has the lowest value when translated to a %age corresponds to the food's DIAAS rating [49]. The digestible indispensable amino acid score has the potential to become increasingly well-known as a criterion for protein quality. The following suggestions based on the DIAAS scale are projected to result in published and widespread protein classifications.

1. A claim will not be associated with a score of less than 75%
2. A score of at least 75% and 99% but less than that denotes “Good Protein Quality”
3. “Excellent Protein Quality” on the DIASS is defined as a score of 100% or higher

Low-quality proteins that the small intestine would often reject can be easily broken down when fecal matter forms inside the large intestine. With this knowledge, the importance of sampling close to small intestine's end is made obvious [92].

## 4. Conclusion

Whey comprises a variety of ingredients, including immunoglobulins, lactoferrin, *β*-lactalbumin, and lactoglobulin which are the main parts of whey proteins and often have a wide range of effects on people. Whey and its derivatives, including lactoglobulins, immunoglobulins, and lactalbumin, have a number of favorable functional properties. The two main whey proteins, *α*-lactalbumin (*α*-la) and *β*-lactoglobulin (*β*-LG), were intact proteins, and the suppression of ACE, anticarcinogenic activity, antimicrobial activity, metabolic hypocholesterolaemia effect, and physiological effects is all documented by peptides produced from proteins. The manufacture of numerous foods, including whey protein, whey drinks, and other food products, is just one of the many uses of whey. One of the most widely used applications is the manufacture or extraction of whey proteins, which are typically found in whey processing sector. In addition, whey-derived bioactive substances such as essential amino acids, IGs, micronutrients, BSA, LP, LF, and GMP have a variety of biological, pharmacological, and therapeutic applications. Since whey boosts the human immune system and provides the body with the nutrients it requires, studies have suggested that it may have a good effect on health. The use of whey has been shown in recent research to aid in the prevention of COVID-19 by improving the body's immune performance.

Researchers are looking for new and more appealing whey-based products with the potential to improve health as a result of the functional qualities of whey. Future research should focus on whey protein development, including standardisation studies and creating strategies to improve the stability of the product's both short- and long-term shelf lives. Food products created with whey should be produced with good manufacturing practices and quality control.

## Figures and Tables

**Table 1:** Concentration of whey protein constituents in cow milk.

Whey protein constituents	Concentration (g/L)	References
*α*-lactalbumin	1.2	[2, 8, 18, 19]
*β*-lactoglobulin	1.3	
Bovine serum	0.4	
Immunoglobulin	0.7	
Glycomacropeptide	1.2	
Proteose-peptones	0.2-0.4	
Lactoferrin	0.1	
Bovine lactoperoxidase	0.03	

**Table 2:** Components and %age of proteins in various milks.

Components	Cow milk	Buffalo milk	Goat milk	Sheep milk	References
Whey protein (g/L)	6.46	6.46	6.14	10.76	[2, 36–38]
*α*-lactalbumin (%)	16.2	16.2	21	10.8	
*β*-lactoglobulin (%)	59.3	58	54	61	
Immunoglobulin (%)	15	15	11.5	20	
Serum albumin/lactoferrin (%)	9.5	9.5	12.8	8.2	

**Table 3:** Amino acid content of various milk.

Amino acids (g/100 g)	Cow milk	Goat milk	Buffalo milk	References
Aspartic acid	7.8	7.4	7.13	[1, 8, 19, 36, 48]
Serine	4.8	7.4	7.13	
Threonine	4.5	5.7	5.7	
Glutamic acid	23.2	19.3	21.4	
Proline	9.6	14.6	12	
Cystine	0.6	0.6	0.58	
Glycine	1.8	2.1	1.9	
Alanine	3	3.5	3.03	
Valine	4.8	5.4	6.7	
Methionine	1.8	3.5	0.9	
Isoleucine	4.5	7	5.7	
Leucine	8.5	8.2	9.5	
Tyrosine	4.3	4.8	3.8	
Phenylalanine	4.8	6.0	4.7	
Histidine	3	5	2.7	
Lysine	8	8	7.3	

**Table 4:** Therapeutic applications of whey protein in humans.

Whey components	Whey protein (%)	Benefits	References
Beta-lactoglobulin	50-55	Source of branched-chain and essential amino acids	[23, 36, 77–79]
Alpha-lactalbumin	20-25	Sources of branched-chain and essential amino acids	
Immunoglobulins	10-15	Immune modulating benefits	
Lactoferrin	1-2	Antibacterial, antiviral, antifungal, and antioxidant properties encourage the development of necessary microorganisms	
Lactoperoxidase	0.5	Inhibits the bacterial growth	
Bovine serum	5-10	A source of vital amino acids hefty protein	
Glycomacropeptide	10-15	Branched-chain amino acid source	

## Data Availability

All the discussed data are available therein the text.
